# Quantifying the dynamic transmission of COVID-19 asymptomatic and symptomatic infections: Evidence from four Chinese regions

**DOI:** 10.3389/fpubh.2022.925492

**Published:** 2022-09-29

**Authors:** Yuanyuan Pei, Yi Guo, Tong Wu, Huiying Liang

**Affiliations:** ^1^Clinical Data Center, Guangzhou Women and Children's Medical Center, Guangdong Provincial Clinical Research Center for Child Health, Institute of Pediatrics, Guangzhou Medical University, Guangzhou, China; ^2^Medical Research Department, Guangdong Provincial People's Hospital/Guangdong Academy of Medical Sciences, Guangzhou, China

**Keywords:** coronavirus disease 2019 (COVID-19), asymptomatic infections, symptomatic infections, dynamic transmission, susceptibility–exposure–infection–recovery (SEIR)

## Abstract

The dynamic transmission of asymptomatic and symptomatic COVID-19 infections is difficult to quantify because asymptomatic infections are not readily recognized or self-identified. To address this issue, we collected data on asymptomatic and symptomatic infections from four Chinese regions (Beijing, Dalian, Xinjiang, and Guangzhou). These data were considered reliable because the government had implemented large-scale multiple testing during the outbreak in the four regions. We modified the classical susceptible–exposure–infection–recovery model and combined it with mathematical tools to quantitatively analyze the number of infections caused by asymptomatic and symptomatic infections during dynamic transmission, respectively. The results indicated that the ratios of the total number of asymptomatic to symptomatic infections were 0.13:1, 0.48:1, 0.29:1, and 0.15:1, respectively, in the four regions. However, the ratio of the total number of infections caused by asymptomatic and symptomatic infections were 4.64:1, 6.21:1, 1.49:1, and 1.76:1, respectively. Furthermore, the present study describes the daily number of healthy people infected by symptomatic and asymptomatic transmission and the dynamic transmission process. Although there were fewer asymptomatic infections in the four aforementioned regions, their infectivity was found to be significantly higher, implying a greater need for timely screening and control of infections, particularly asymptomatic ones, to contain the spread of COVID-19.

## Introduction

The novel coronavirus disease 2019 (COVID-19) pandemic poses a major threat to human health worldwide (1). Several studies have found that asymptomatic infections exacerbate the prevalence of COVID-19 (2, 3). In this study, according to The Novel Coronavirus Pneumonia Prevention and Control Plan (Seventh Edition), “asymptomatic infections” refers to persons infected with SARS-CoV-2 but who have never experienced symptoms and persons infected who had no symptoms at first but developed symptoms later, both the groups are infectious, and SARS-CoV-2 can harm their bodies (4). Previous studies have shown that the load of severe acute respiratory syndrome coronavirus 2 (SARS-CoV-2) in asymptomatic infections is similar to that of symptomatic infections; however, the former demonstrated longer viral ribonucleic acid (RNA) shedding times and weaker immune responses (5, 6). To curb the spread of COVID-19, prevention and control measures include vaccination, universal mask-wearing, reduced social interaction, virus testing, and social isolation (7, 8). Nevertheless, severe prevention and control measures have caused widespread debate and discontent, negatively affecting economies and social activities (9). Hence, quantitative analysis of the impact of symptomatic and asymptomatic infections on the pandemic is essential for formulating reasonable epidemic prevention measures.

As asymptomatic infections are not easily detected because of their insidious nature, a second pandemic may occur if asymptomatic infections are not detected after symptomatic cases have been controlled (10). Several previous studies of transmission efficacy in asymptomatic infections were based on follow-ups of infected individuals. Hoxha et al. (11) found 6,244 asymptomatic infections out of 8,343 patients who tested positive (74.8%) in the analysis of an extensive testing in Belgium. Bi et al. (12) found that 25 patients (6%) were asymptomatic infections in a retrospective cohort study of 391 cases in Shenzhen. A cohort study of 628 SARS-CoV-2 positive patients and 3,790 close contacts by Sayampanathan et al. (13) showed that symptomatic infections led to 3.85 times more infections in close contact than asymptomatic infections. The US Centers for Disease Control and Prevention estimated that 35% of SARS-CoV-2 infections were asymptomatic, and 40% of the virus transmission occurred prior to the onset of symptoms (14). The wide variation in results across studies presents a challenge to understanding the transmission of this disease. Moreover, the above follow-up studies tend to miss asymptomatic infections and are laborious and time-consuming.

To study the rampant spread of COVID-19, various analytical methods and artificial intelligence modeling have been used and effective results have been achieved. Mahmoudi et al. (15) studied the relationship between the transmission rates of COVID-19 in high-risk countries by principal component analysis. Deif et al. (16) identified SARS-CoV-2 from viral genome sequences using deep bidirectional recurrent neural networks. Kumar et al. (17) achieved the prediction of COVID-19 using a recurrent neural network and reinforcement learning model. Due to the lack of adequate vaccines or effective therapeutic drugs, mathematical models were used to analyze, predict, and develop non-pharmacological interventions. Sarkar et al. (18), that modeled and predicted the COVID-19 pandemic in India. Khajanchi et al. (19) studied mathematical models and intervention strategies for the COVID-19 outbreak. Khajanchi and Sarkar (20) also predicted the daily and a cumulative number of cases of the COVID-19 pandemic in India. Samui et al. (21) used a dynamic model to study the spread of COVID-19. In addition, some key factors influencing the COVID-19 pandemic, such as media, were analyzed by the extended susceptibility–exposure–infection–recovery (SEIR) model (22, 23). The basic reproductive number R0 is one of the most critical parameters in the study of infectious diseases (24). For R0 < 1, the transmission of the infectious disease is expected to stop; for R0 = 1, an infected individual can infect one person on average, that is, the spread of the disease is stable; for R0 > 1, the transmission of the infectious disease becomes epidemic. The aforementioned challenges and findings prompted us to study the dynamic transmission of asymptomatic infections.

In this study, we sought to modify the classical SEIR epidemiological model and combine it with mathematical tools to quantitatively analyze and compare the impact of asymptomatic and symptomatic infections on the COVID-19 pandemic and their transmission dynamics.

## Data and methods

### Data sources

Detecting asymptomatic infections requires large-scale, multiple, intensive reverse transcription-polymerase chain reaction (RT-PCR) tests (25, 26). Since the initial COVID-19 outbreak in Wuhan, the government of China has strengthened pandemic prevention and control measures. On January 28, 2020, China's National Health and Wellness Commission issued the Novel Coronavirus Pneumonia Diagnosis and Treatment Protocol (Third Edition) (27). This included data on asymptomatic infections in prevention and control management, requiring health care institutions at all levels to directly report if they detected asymptomatic infections within 2 h *via* the internet. After receiving reports of asymptomatic infections, disease control agencies at county and district levels were tasked to complete case investigations within 24 h and promptly register and report close contacts.

From April 1, 2020, the National Health Commission of China published data on asymptomatic infections and referrals in the daily pandemic notification system (“referrals” meant infectious persons who were asymptomatic at first, but developed symptoms later). The Chinese government organized and conducted multiple large-scale RT-PCR tests in the regions of known infections. Thus, the publicly available numbers of asymptomatic and symptomatic infections were reliable. For example, Guangzhou experienced a COVID-19 outbreak in May 2021, and 27,985,500 RT-PCR tests were performed cumulatively in the region (28).

**Figure 1:**
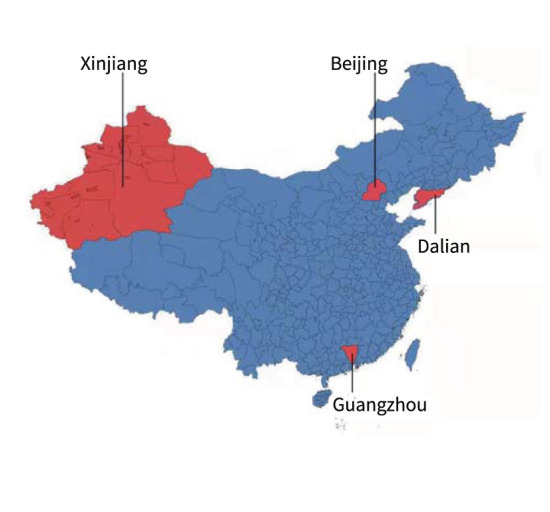
Outbreaks in Beijing, Dalian, Xinjiang, and Guangzhou.

**Figure 2:**
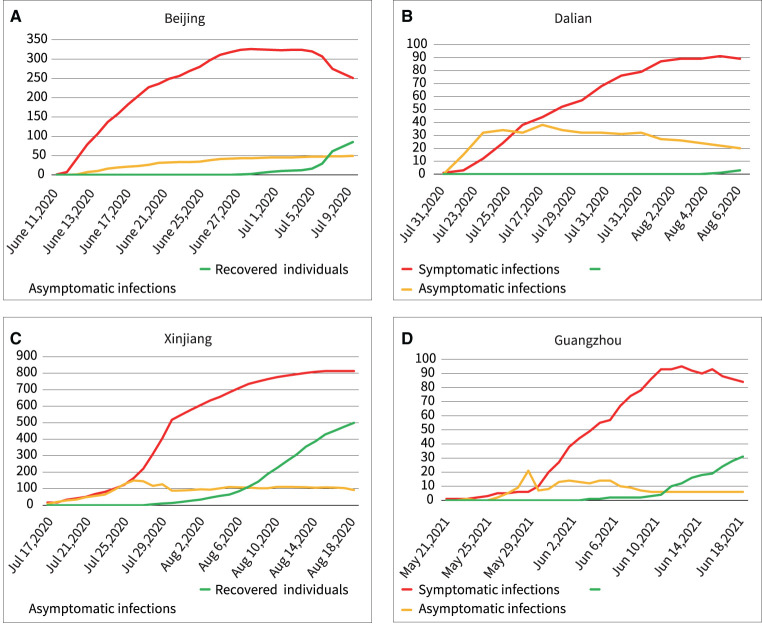
Outbreaks in the four regions include asymptomatic infections and symptomatic infections, and recovered individuals. **(A)** Beijing, **(B)** Dalian, **(C)** Xinjiang, **(D)** Guangzhou.

After the Wuhan outbreak, during the period until July 2021, four other outbreaks occurred in China Beijing, Dalian, Xinjiang, and Guangzhou. We modeled and quantitatively analyzed these four outbreaks based on publicly available data (including symptomatic and asymptomatic infections and recovered individuals). We extracted daily outbreak data in Beijing (June 11–July 10, 2020), Dalian (July 22–August 6, 2020), Xinjiang (July 17–August 18, 2020), and Guangzhou (May 21–June 19, 2020) from the following publicly available websites: http://wjw.beijing.gov.cn/wjwh/ztzl/xxgzbd/, https://www.dl.gov.cn/col/col459/index.html, http://wjw.xinjiang.gov.cn/, and http://wjw.gz.gov.cn/ztzl/xxfyyqfk/. The start date was when the first case of infection was detected in the area; the end date was when the last case of infection was detected. The data included the total population of the region, geographical location, daily number of existing symptomatic and asymptomatic infections, and daily number of recovered persons, as shown in Figures 1, 2. We obtained the ratios of the total number of asymptomatic to symptomatic infections: 0.13:1, 0.48:1, 0.29:1, and 0.15:1 in Beijing, Dalian, Xinjiang, and Guangzhou, respectively. All data of the patients were de-identified; therefore, no written informed consent and ethical approval were required.

### Combining mathematical tools to develop an extended SEIR model

**Figure 3:**
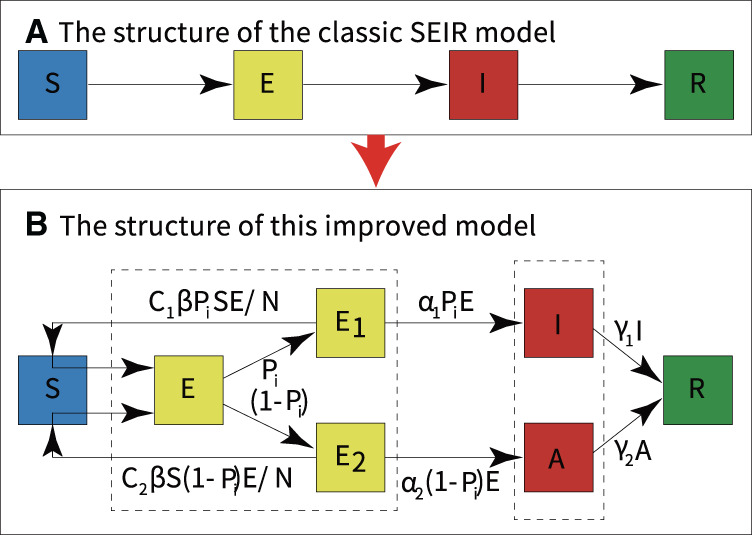
Modifications to the classic SEIR model. **(A)** The structure of the classic SEIR model. **(B)** The structure of this modified SEIR model.

The SEIR model assumes the same probability of exposure for a population in a confined space, and the transmission pattern of an infectious disease over time is assumed to occur in the four states: susceptible, exposed, infectious, and recovered. The parameters of the model control the rate of transition of the population from susceptible, exposed, and infected to recovered individuals. Infectious diseases and various epidemic prevention measures interact dynamically, and the parameters of the model are allowed to change over time and region. The SEIR model is used to study the transmission speed, spatial range, transmission route, and dynamic mechanism of infectious diseases and provide guidance on the effective prevention and control measures of infectious diseases (29, 30). We developed a new SEIR model by modifying the basic and widely used SEIR model and combining it with mathematical tools, as shown in Figure 3.

**Figure 4:**
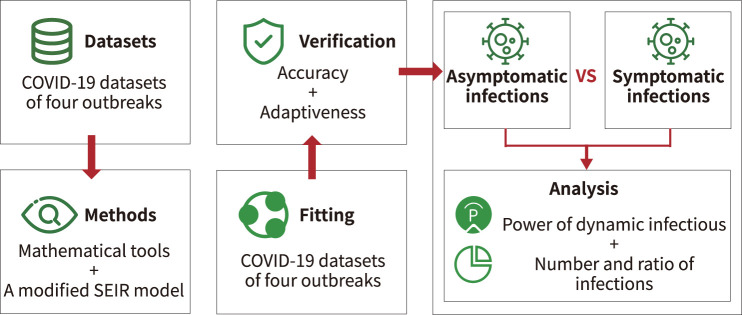
Experimental flow.

The model classified infections into two categories: asymptomatic infections and symptomatic infections. According to the literature, we set upper and lower bounds for each parameter of the model (31–33). Then, historical real epidemic data of the outbreak region were automatically fitted with the new SEIR model. Moreover, the optimal set of parameters for the model was automatically determined by combining the standard variance function and the optimal loss function. Finally, quantitative analysis of symptomatic and asymptomatic infections was performed using the model with the new parameters, the experimental flow is shown in Figure 4.

To model the dynamic transmission of COVID-19, we divided the population in the outbreak regions into different categories:

- S: Healthy and never-infected people who lack immunity and are susceptible to infection.
- E: Contact infection, transmission of infection, and possible conversion to undetected symptomatic or completely asymptomatic infections.
- E1: Contact infection, transmission of infection, and possible conversion to undetected symptomatic infections.
- E2: Contact infection, transmission of infection, and possible conversion to undetected asymptomatic infections.
- I: Infected persons with symptoms who can transmit the virus to S and turn S into E1 or E2 or I or A.
- A: Infected persons who are asymptomatic but can transmit the virus to S and turn S into E1 or E2 or I or A.
- R: People who recover from symptomatic and asymptomatic infections who recover their immunity and do not reconvert to E1 or E2 or I or A.
- N: The total number of people in a region held constant, excluding newborns, immigrants, and deaths.

In the modified SEIR model, each category is dynamically transformed, as shown in Figure 3B. The dynamic transformation relationship forms an ordinary differential equation in each category. The equations of the new model are as follows, and the parameters of the model are described in Table 1.


(1) $$\begin{array}{l} \frac{dS}{dt}=-\frac{C_{1}\beta SP_{i}E}{N}-\frac{C_{2}\beta S\left(1-P_{i}\right)E}{N} \end{array}$$

(2) $$\begin{array}{l} \frac{dE}{dt}=\frac{C_{1}\beta SP_{i}E}{N}+\frac{C_{2}\beta S\left(1-P_{i}\right)E}{N}-\alpha_{1}P_{i}E-\alpha_{1}\left({1-P}_{i}\right)E \end{array}$$

(3) $$\begin{array}{l} \frac{dI}{dt}=\alpha_{1}P_{i}E-\gamma_{1}I \end{array}$$

(4) $$\begin{array}{l} \frac{dA}{dt}=\alpha_{2}\left(1-P_{i}\right)E-\gamma_{2}A \end{array}$$

(5) $$\begin{array}{l} \frac{dR}{dt}=\gamma_{1}I+\gamma_{2}A \end{array}$$

(6) $$\begin{array}{l} N=S+E+I+A+R \end{array}$$

Note that Equation (1) represents the rate of continuous removal from the susceptible persons (S). During the pandemic, people who converted to E1 or E2 were continuously removed from S; thus, the rate of both was <0. Similarly, Equations (2), (3), and (4) can also be understood. In Equation (5), the rate of recovery for symptomatic and asymptomatic infections per day is indicated. To understand these equations, the literature can also be consulted (34).

**Table 1:** Description of the model's parameters.

**Parameters**	**Implication**
C_1_	C_1_ is the average daily number of contacts for each person in E1.
C_2_	C_2_ is the average daily number of contacts for each person in E2.
β	The infection rate of symptomatic and asymptomatic infections. If on average, an infected person is exposed to N persons, and the probability of infection after exposure is P (0–1), β= N*P.
P_i_	The conversion ratio from E to E1.
α_1_	Conversion parameters from E_1_ to I [Countdown of the incubation period (5.2 days) for symptomatic infections].
α_2_	Conversion parameters from E_2_ to A [Countdown of the incubation period (5.2 days) for asymptomatic infections].
γ_1_	The proportion of recovered persons among symptomatic infected persons [Countdown of recovery time (14 days) for symptomatic infections].
γ_2_	The proportion of recovered persons among asymptomatic infected persons [Countdown of recovery time (14 days) for asymptomatic infections].
Decays	Decay constants for transmission parameters.
t	Days.

The parameters in Table 1 (C1, C2, β, Pi, α_1_, α_2_, γ_1_, γ_2_, *decays*) have different values in the different outbreak regions, depending on the detection rates, social distance conditions, vaccination rates, types of treatment, and other control measures.

### Training and fitting on historical data to obtain optimal parameter set

Manually adjusting the parameters of the model to fit real historical datasets is a rather tedious task and can lead to difficulties in obtaining an optimal fit. In this study, the upper and lower limits of the parameters were set according to the references. Subsequently, mathematical tools and the extended SEIR model were used to fit the datasets while obtaining an optimal set of parameters. The process is described below.

First, we set the parameter range. Because the virus was still mutating, different regions had different epidemiological characteristics; moreover, the pandemic developed dynamically. As a result of a number of factors, including varying population sizes, densities, and the use of epidemic prevention measures, multiple factors influenced the progression of the pandemic. Therefore, the model parameters were different in different regions. The values of some initial parameters were based on references (31–35), the upper and lower limits of “β” and “decays” were set to 0.1–0.8 and 0-∞, respectively. The E to I transfer rate was the reciprocal of the incubation period, which was reported to have a mean value of 5.2 d, the “α1” and “α2” were the reciprocal of 5.2 d, hence, the upper and lower limits were set to 0.16–0.3. The recovery time for infections was 14 d, “γ1” and “γ2” were the reciprocal of the recovery time (14 d) for symptomatic and asymptomatic infections, thus, the upper and lower limits were set to 0.06–0.1. Because the asymptomatic infections were not easily detected, the transmission to healthy individuals was higher than that of symptomatic infections (36). In the study by Yang et al. (37), the number of close contacts during the latency period was set to three for symptomatic infections and 15 for asymptomatic infections, therefore, the upper and lower limits of “c1” and “c2” were set to 0–0.6 and 0.9–4, respectively (2, 12, 38). Furthermore, the upper and lower limits of the total number of people were set to 1e3–1e7, depending on the size of the infection outbreak area. Here, “close contact” was defined as unprotected, close contact within 1 m. The upper and lower limits of the parameter set are shown in Table 2.

**Table 2:** Upper and lower limit interval settings for model parameters.

**Name**	**c_1_**	**c_2_**	**P_i_**	**β**	**α_1_**	**α_2_**	**γ_1_**	**γ_2_**	**N0**	**Decays**
Lower	0	0.9	0.2	0.1	0.16	0.16	0.06	0.06	1e3	0
Upper	0.6	4	0.8	0.8	0.3	0.3	0.1	0.1	1e7	∞

Second, when programming with MATLAB R2018b (https://ww2.mathworks.cn/en/), the standard variance loss function was used to describe the deviation between the data obtained from the model and the real data of the pandemic, as shown in Equation (7).


(7) $$\begin{array}{l} Loss\left(t\right)=\overset{t=end}{\underset{t=start}{\sum }}{\left(I_{\left(fit\right)}t-I_{\left(real\right)}t\right)}^{2}+{\left(A_{\left(fit\right)}t-A_{\left(real\right)}t\right)}^{2} \end{array}$$

After obtaining the minimum deviation, to obtain a parameter set that fits well with the real scenario data, we combined the MATLAB function “fmincon” which automatically finds and determines the optimal value in a set of parameters with upper and lower limits, as shown in Equation (8).


(8) $$\begin{array}{l} \begin{array}{l} Para\;=\;fmincon\;(@seir.loss,para,\;\left[\;],[\;],[\;\right],\;lb,\;ub) \end{array} \end{array}$$

Therefore, the optimal fit of the model was obtained, as well as the optimal parameters to match the pandemic transmission characteristics of the region.

### Experimental environments

All experiments were performed on a personal computer with Windows 7 Home Edition 64-bit operating system, and the analysis software was MATLAB R2018b (https://ww2.mathworks.cn/en/) and SPSS version 26 (IBM Corp., Chicago, IL, USA).

## Results

We used real scene data of COVID-19 outbreaks from four regions (Beijing, Dalian, Xinjiang, and Guangzhou) in China and ran the data on the new model. The following results were obtained.

### Fitting results of the four outbreaks

**Figure 5:**
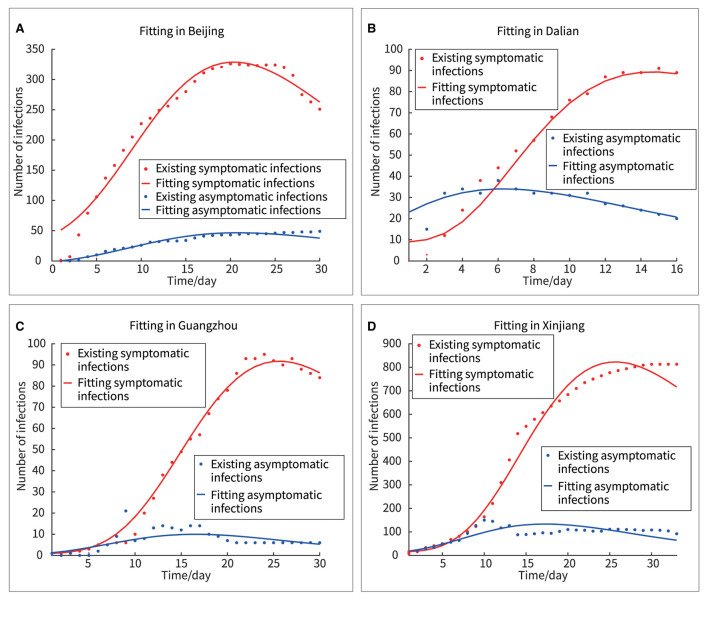
Fitting of four outbreaks of symptomatic and asymptomatic infections. **(A)** Beijing, **(B)** Dalian, **(C)** Guangzhou, **(D)** Xinjiang.

Fitting the new model to outbreak data (both symptomatic and asymptomatic infections) in Beijing, Dalian, Xinjiang, and Guangzhou yielded the results shown in Figure 5.

In Figure 5, the red and blue dots represented the real asymptomatic and symptomatic infections, respectively, and the red and blue curves represented the fitted curves of the new model for asymptomatic and symptomatic infections, respectively. Here, two methods, “F-test” and “Goodness of Fit” were used to analyze the differences between the real and fitted datasets.

First, using SPSS version 26, the difference between the real and fitted datasets was obtained by F-test, as shown in Table 3, with all F<0.2, and all sig>0.6, proving that there was no significant difference between the real and fitted datasets; the new model had good fitting ability in all four regions.

**Table 3:** Analysis of the “F-test” in symptomatic and asymptomatic infections.

**No**	**Subject**	**SS**	**DF**	**MS**	**F**	**SIG**
1	BJ-F-ASyma	0.384	1.000	0.384	0.002	0.969
2	BJ-F-Syma	121.166	1.000	121.166	0.013	0.910
3	DL-F-Asyma	1.511	1.000	1.511	0.020	0.890
4	DL-F-Syma	12.139	1.000	12.139	0.012	0.914
5	XJ-F-Asyma	10.712	1.000	10.712	0.010	0.921
6	XJ-F-Syma	346.763	1.000	346.763	0.004	0.953
7	GZ-F-Asyma	2.977	1.000	2.977	0.190	0.665
8	GZ-F-Syma	11.515	1.000	11.515	0.009	0.927

BJ-F-ASyma, The “F-Test” for asymptomatic infections in Beijing; BJ-F-Syma, The “F-Test” for symptomatic infections in Beijing; DL-F-ASyma, The “F-Test” for asymptomatic infections in Dalian; DL-F-Syma, The “F-Test” for symptomatic infections in Dalian; XJ-F-ASyma, The “F-Test” for asymptomatic infections in Xinjiang; XJ-F-Syma, The “F-Test” for symptomatic infections in Xinjiang; GZ-F-ASyma, The “F-Test” for asymptomatic infections in Guangzhou; GZ-F-Syma, The “F-Test” for symptomatic infections in Guangzhou; SS, Sum of Squares; DF, Degrees of Freedom; MS, Mean Square; F, F-Value; and SIG, Significance.

Second, we evaluated the “Goodness of Fit” of the model using the coefficient of determination R^2^, which was expressed by the following equation (ŷ_*i*_ represented the predicted value of each point and $\bar{y}$ the average value of each point).


(9) $$\begin{array}{l} R^{2}=\frac{\sum_{i=1}^{n}{\left({\hat{y}}_{i}-\bar{y}\right)}^{2}}{\sum_{i=1}^{n}{\left(y_{i}-\bar{y}\right)}^{2}} \end{array}$$

**Figure 6:**
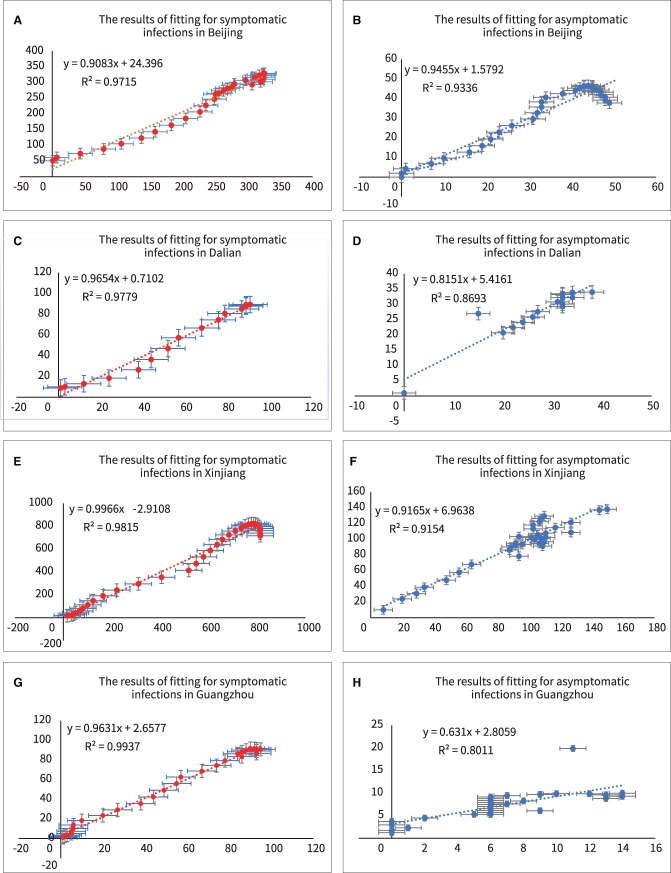
Analyzing of fitting for symptomatic and asymptomatic infections in four outbreaks. **(A)** R^2^ of symptomatic infections in Beijing, **(B)** R^2^ of asymptomatic infections in Beijing, **(C)** R^2^ of symptomatic infections in Dalian, **(D)** R^2^ of asymptomatic infections in Dalian, **(E)** R^2^ of symptomatic infections in Xinjiang, **(F)** R^2^ of asymptomatic infections in Xinjiang, **(G)** R^2^ of symptomatic infections in Guangzhou, **(H)** R^2^ of asymptomatic infections in Guangzhou.

The closer each observation was to the trend line, the better the fit was implied. The results were shown in Figure 6 and Table 4. The R^2^ values for both symptomatic and asymptomatic infections in all four regions were close to 1, indicating a good fit of the model to the actual data (R^2^ values >0.8 usually indicated a good fit).

**Table 4:** Analysis of the “Goodness of Fit” in symptomatic and asymptomatic infections.

**No**	**Region**	**FitASym^a^**	**FitSym^b^**
1	BJ	93.36%	97.15%
2	DL	86.93%	97.79%
3	XJ	91.54%	98.15%
4	GZ	80.11%	99.37%

BJ, Beijing; DL, Dalian; XJ, Xinjiang; and GZ, Guangzhou; FitASym^a^, R^2^ of asymptomatic infections; FitSym^b^, R^2^ of symptomatic infections.

### Power of dynamic transmission of symptomatic and asymptomatic infections

In this model, c1 and β interact with each other and yield an overall dynamic transmitted power. The dynamic transmission power parameters of symptomatic and asymptomatic infections were expressed by Equations (10) and (11), respectively. “Syinfect” represented the dynamic transmission power parameters of symptomatic infections, and “Asyinfect” represented the dynamic transmission power parameters of asymptomatic infection.


(10) $$\begin{array}{l} Syinfect\left(t\right)=c_{1}\times \beta \times e^{-decays\times t}\times P_{i} \end{array}$$

(11) $$\begin{array}{l} Asyinfect(t)=c_{2}\times \beta \times e^{-decays\times t}\times \left(1-P_{i}\right) \end{array}$$

Figure 6 showed the dynamic transmission power curves for asymptomatic and symptomatic infections. Their initial dynamic transmission power parameters in Beijing, Dalian, Xinjiang and Guangzhou were 1.15:0.25, 1.30:0.20, 0.48:0.32, and 0.55:0.31, respectively. It was shown that the initial dynamic transmission power parameters were lasrger for asymptomatic infections than for symptomatic infections. Furthermore, larger initial transmission power parameters predicted stronger subsequent outbreaks. Under effective control measures, the dynamic transmission power parameter would keep decreasing, and when the dynamic transmission power parameter equaled zero, it indicated the end of the outbreak.

The mathematical integrals of the dynamic transmission power parameter curves for asymptomatic and asymptomatic infections were represented by Equations (12) and (13), respectively. “Sy” represented the integration under the curve of dynamic transmission power parameters for symptomatic infections, and “Asy” represented the integration under the curve of dynamic transmission power parameters for asymptomatic infections.


(12) $$\begin{array}{l} Sy\left(t\right)=\int_{start}^{end}I\left(t\right)dt \end{array}$$

(13) $$\begin{array}{l} Asy\left(t\right)=\int_{start}^{end}I\left(t\right)dt \end{array}$$

**Figure 7:**
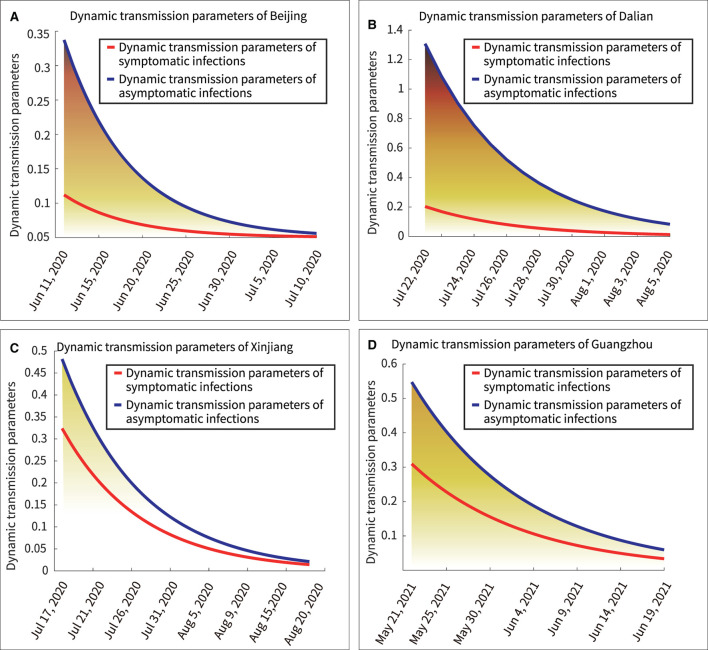
Dynamic transmission power parameters of symptomatic and asymptomatic infections. **(A)** Beijing, **(B)** Dalian, **(C)** Xinjiang, **(D)** Guangzhou.

The area under the curve represented the total transmission power. The results for the total transmission power of asymptomatic and symptomatic infections in the entire outbreak were as follows: Beijing, 9.05:1.95; Dalian, 7.37:1.15; Xinjiang, 4.97:3.34; and Guangzhou, 6.69:3.78. For example, the ratio of the area under the blue curve to the area under the red curve in Figure 7A is 9.05:1.95. This indicated that the asymptomatic infections were more infectious than the symptomatic infections in above four outbreaks.

### Number of people infected by symptomatic or asymptomatic infections

According to the model, the number of people infected by asymptomatic and symptomatic infections per day was expressed in Equations (14) and (15), respectively. “NoaSya” represented the number of healthy people infected by asymptomatic infections per day, and “Nosyb” represented the number of healthy people infected by symptomatic infections per day. Table 5 showed the results.


(14) $$\begin{array}{l} NoaSyaN\left(t\right)=\frac{c_{2}\times \beta \times e^{-decays\times t}\times (1-P_{i})\times S\times E}{N} \end{array}$$

(15) $$\begin{array}{l} Nosyb\left(t\right)=\frac{c_{1}\times \beta \times e^{-decays\times t}\times P_{i}\;\times S\times E}{N} \end{array}$$

**Figure 8:**
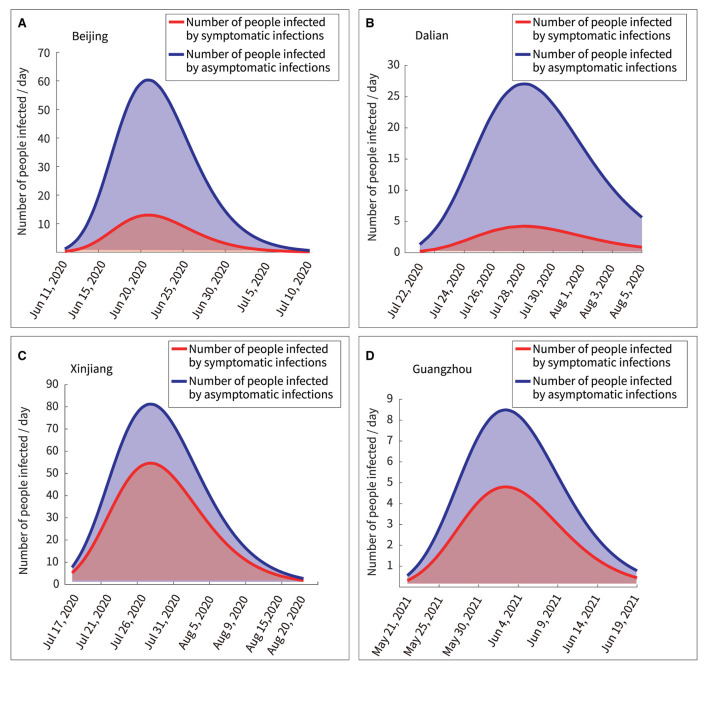
Quantitative analysis of persons infected by symptomatic and asymptomatic infections. **(A)** Beijing, **(B)** Dalian, **(C)** Xinjiang, **(D)** Guangzhou.

The curves of Equations (14) and (15) were mathematically integrated separately to obtain the area under the curve, as shown in Figure 8. The ratios of the total number of asymptomatic infections to the total number of asymptomatic infections obtained as 4.64:1, 6.21:1, 1.49:1, and 1.76:1 in Beijing, Dalian, Xinjiang, and Guangzhou, respectively. Thus, the following two points could be summarized.

1. Quantitative analysis by the transmission dynamics equations of the model showed the transmission process of asymptomatic and symptomatic infections in healthy persons, respectively. Further, the analysis of the model was more convenient than following up infections in a large population.
2. The number of asymptomatic infections was lower than the number of symptomatic infections; however, asymptomatic infections spread the disease to a larger number of people.

**Table 5:** The number of daily infections caused by asymptomatic and symptomatic infections.

**No**.	**Region**	**Date**	**NoaSy^a^**	**Nosy^b^**	**No**.	**Region**	**Date**	**NoaSy^a^**	**Nosy^b^**
1	BJ	2020/6/11	1	0	56	XJ	2020/7/26	78	52
2	BJ	2020/6/12	3	1	57	XJ	2020/7/27	81	54
3	BJ	2020/6/13	6	1	58	XJ	2020/7/28	81	55
4	BJ	2020/6/14	12	3	59	XJ	2020/7/29	80	54
5	BJ	2020/6/15	19	4	60	XJ	2020/7/30	77	52
6	BJ	2020/6/16	30	6	61	XJ	2020/7/31	72	48
7	BJ	2020/6/17	39	9	62	XJ	2020/8/1	66	45
8	BJ	2020/6/18	48	10	63	XJ	2020/8/2	60	40
9	BJ	2020/6/19	55	12	64	XJ	2020/8/3	53	36
10	BJ	2020/6/20	60	13	65	XJ	2020/8/4	46	31
11	BJ	2020/6/21	61	13	66	XJ	2020/8/5	40	27
12	BJ	2020/6/22	59	13	67	XJ	2020/8/6	34	23
13	BJ	2020/6/23	55	12	68	XJ	2020/8/7	29	19
14	BJ	2020/6/24	49	10	69	XJ	2020/8/8	24	16
15	BJ	2020/6/25	42	9	70	XJ	2020/8/9	20	13
16	BJ	2020/6/26	36	8	71	XJ	2020/8/10	16	11
17	BJ	2020/6/27	29	6	72	XJ	2020/8/11	13	9
18	BJ	2020/6/28	24	5	73	XJ	2020/8/12	11	2
19	BJ	2020/6/29	19	4	74	XJ	2020/8/13	9	6
20	BJ	2020/6/30	14	3	75	XJ	2020/8/14	7	5
21	BJ	2020/7/1	11	2	76	XJ	2020/8/15	5	4
22	BJ	2020/7/2	8	2	77	XJ	2020/8/16	4	3
23	BJ	2020/7/3	6	1	78	XJ	2020/8/17	3	2
24	BJ	2020/7/4	5	1	79	XJ	2020/8/18	3	2
25	BJ	2020/7/5	3	1	80	GZ	2021/5/21	1	0
26	BJ	2020/7/6	2	1	81	GZ	2021/5/22	1	1
27	BJ	2020/7/7	2	0	82	GZ	2021/5/23	1	1
28	BJ	2020/7/8	1	1	83	GZ	2021/5/24	2	1
29	BJ	2020/7/9	1	0	84	GZ	2021/5/25	3	2
30	BJ	2020/7/10	1	0	85	GZ	2021/5/26	4	2
31	DL	2020/7/22	1	0	86	GZ	2021/5/27	5	3
32	DL	2020/7/23	3	1	87	GZ	2021/5/28	6	3
33	DL	2020/7/24	7	1	88	GZ	2021/5/29	6	4
34	DL	2020/7/25	12	2	89	GZ	2021/5/30	7	4
35	DL	2020/7/26	18	3	90	GZ	2021/5/31	8	4
36	DL	2020/7/27	23	4	91	GZ	2021/6/1	8	5
37	DL	2020/7/28	26	4	92	GZ	2021/6/2	9	5
38	DL	2020/7/29	27	4	93	GZ	2021/6/3	8	5
39	DL	2020/7/30	26	4	94	GZ	2021/6/4	8	5
40	DL	2020/7/31	24	4	95	GZ	2021/6/5	8	4
41	DL	2020/8/1	21	3	96	GZ	2021/6/6	7	4
42	DL	2020/8/2	17	3	97	GZ	2021/6/7	7	4
43	DL	2020/8/3	13	2	98	GZ	2021/6/8	6	3
44	DL	2020/8/4	10	2	99	GZ	2021/6/9	5	3
45	DL	2020/8/5	8	1	100	GZ	2021/6/10	5	2
46	DL	2020/8/6	6	1	101	GZ	2021/6/11	4	2
47	XJ	2020/7/17	8	5	102	GZ	2021/6/12	3	2
48	XJ	2020/7/18	13	9	103	GZ	2021/6/13	3	2
49	XJ	2020/7/19	19	13	104	GZ	2021/6/14	2	1
50	XJ	2020/7/20	27	18	105	GZ	2021/6/15	2	1
51	XJ	2020/7/21	36	24	106	GZ	2021/6/16	2	1
52	XJ	2020/7/22	46	31	107	GZ	2021/6/17	1	1
53	XJ	2020/7/23	56	38	108	GZ	2021/6/18	1	1
54	XJ	2020/7/24	65	44	109	GZ	2021/6/19	1	0
55	XJ	2020/7/25	72	49	…	…	…	…	…

BJ, Beijing; DL, Dalian; XJ, Xinjiang; and GZ, Guangzhou.

## Discussion

This study elucidated the ratio of healthy persons infected by asymptomatic and symptomatic infections in a pandemic, and quantified their dynamic transmission processes respectively. Owing to the strict control of the pandemic in China, the proportion of asymptomatic infections was smaller than that of symptomatic infections in the four outbreaks studied. Nevertheless, quantitative comparative analyses confirmed that transmission from asymptomatic infections to healthy individuals was greater than that from symptomatic infections. We searched PubMed for relevant articles in English, published since the database inception before July 25, 2021, using the search terms (“asymptomatic” [Title/Abstract] AND (“COVID-19” [Title/Abstract] OR “SARS-CoV-2” [Title/Abstract]) AND (“transmit^*^” [Title/Abstract] OR “infect” [Title/ Abstract]) AND (“ratio” [Title/Abstract] OR “proportion” [Title/Abstract])). We identified 144 articles and found six articles that discussed the risk of asymptomatic and symptomatic infections during the COVID-19 pandemic (39–43). Moreover, our study clearly analyzed the dynamic transmission characteristics of asymptomatic and symptomatic COVID-19 infections. Furthermore, this is a multicenter study. Thus, it was proven that the proposed model can be used in different outbreak regions by training and fitting.

Furthermore, there are several findings. First, Figure 7 showed a rapid reduction in dynamic transmission parameters for both symptomatic and asymptomatic infections in the same region, confirming that the epidemic control measures were effective at that time. Second, Figure 2 showed that the proportion of asymptomatic infections was only a small proportion of the overall number of infected individuals. However, Figure 6 showed that the initial transmission dynamic power parameter of asymptomatic infections was larger than that of symptomatic infections. Furthermore, Figure 8 showed that the overall number and ratio of asymptomatic infections transmitted to the healthy persons were greater than those of symptomatic infections throughout the above four outbreaks.

Third, by running the results of the same model on the four outbreaks, we found that the dynamic transmission power parameters of symptomatic and asymptomatic infections differed in different geographical regions, as did the number and proportion of infections in the healthy populations. This explained why previous literature had different ratios of asymptomatic to symptomatic infections in different regions (13–16). Specifically, they were caused by the transmission of different viruses in different populations under different prevention and control measures (32).

Fourth, the results of the data analysis in Figures 7, 8 suggested that the severity of pandemic transmission could be assessed by observing the dynamic transmission parameters of daily asymptomatic infections and asymptomatic infections, and could also be used to assess the effectiveness of control measures. A similar conclusion was reached in a previous study (44).

The results showed that a comparative analysis of the transmission dynamics of symptomatic and asymptomatic infections could provide a reference for highlighting certain measures in the combination of various preventive measures to reduce economic losses. With widespread awareness of COVID-19 and increased vigilance, subjects with symptomatic infection could more likely self-identify and receive treatment, even with mild symptoms. Therefore, the proportion of transmission from asymptomatic infections is likely to continue increasing. Thus, among various epidemic control measures, RT-PCR testing should be enhanced in the outbreak affected region, and isolation treatment for asymptomatic infections should be emphasized.

This study has some limitations. First, there was a time lag between individual-based exposure, symptom onset, and testing confirmation. Furthermore, in China, owing to strict epidemic control measures, patients treated in isolation had to be observed for a period after actual recovery before being declared cured, thereby possibly causing deviations. The data used in our model included the above time lag, therefore, there may be a small deviation in the results, which could explain why the R^2^ was 80.11% after fitting the asymptomatic infections in Guangzhou, although an R^2^ >80% means a good fit. Second, as all patients who tested positive using RT-PCR in China were offered free isolation treatment, this study assumed that patients confirmed by testing were not capable of transmitting the virus during treatment; Third, the upper and lower bounds for the model parameters were set using data from other references. We may not have considered extreme cases, such as some “super spreader” incidents of COVID-19 (45). Fourth, the amount of data was limited, which might have affected the accuracy of the results. Despite the new model having some limitations, the epidemiological data and experimental results were within acceptable limits under the assumption of common sense.

## Conclusion

This multicenter, retrospective study demonstrated the dynamic process of symptomatic and asymptomatic infection transmission to healthy individuals. Although there were fewer asymptomatic infections than symptomatic ones in the four outbreaks studied, a greater number and proportion of infections in the healthy population were caused by asymptomatic infections. These findings suggest that isolation and treatment of symptomatic infections are not sufficient under the conditions of inadequate vaccines, continuous virus mutation, and lack of widespread use of therapies that could eliminate current infectiousness. There is thus a great need to reduce the risk of transmission of asymptomatic infections. On the one hand, in COVID-19 pandemic regions, there is a need for proactive, large-scale, and multiple testing of close contacts of COVID-19 patients to help in mitigating the disease spread and exposure of populations and to rapidly identify and treat asymptomatic infections. On the other hand, healthy people should continue social distancing, wearing masks, and vaccination to avoid infection. The proposed model elucidated the impact of symptomatic and asymptomatic infections on epidemics. The model can also be used to observe the progress of the epidemic and assess the effectiveness of interventions, which could help governments worldwide develop reasonable measures to fight the COVID-19 pandemic.

## Data availability statement

The datasets presented in this study can be found in online repositories. The names of the repository/repositories and accession number(s) can be found in the article/supplementary material.

## Ethics statement

Ethical approval was not provided for this study on human participants because all the data of human participants were de-identified, therefore, the requirement for written informed consent and ethical approval was waived. All procedures performed in studies involving human participants were according to the National Research Committee and the 1964 Helsinki declaration and its later amendments and comparable ethical standards. Written informed consent from the participants' legal guardian/next of kin was not required to participate in this study on the basis of the national legislation and the institutional requirements.

## Author contributions

YP: conceptualization and design, data analysis and proofreading, coding programming, experiment, visualization, and writing–original draft and editing. YG and TW: data searching and proofreading. HL: supervision and guidance. All authors read and approved the manuscript.

## Funding

This work was supported by the Science and Technology Planning Project of Guangdong Province (Approval No. 2020B1111170001).

## Conflict of interest

The authors declare that the research was conducted in the absence of any commercial or financial relationships that could be construed as a potential conflict of interest.

## Publisher's note

All claims expressed in this article are solely those of the authors and do not necessarily represent those of their affiliated organizations, or those of the publisher, the editors and the reviewers. Any product that may be evaluated in this article, or claim that may be made by its manufacturer, is not guaranteed or endorsed by the publisher.
